# Imaging and histopathologic correlates of plasma cell-free DNA concentration and circulating tumor DNA in adult patients with newly diagnosed glioblastoma

**DOI:** 10.1093/noajnl/vdaa016

**Published:** 2020-02-27

**Authors:** Seyed Ali Nabavizadeh, Jeffrey B Ware, Samantha Guiry, MacLean P Nasrallah, Jazmine J Mays, Jacob E Till, Jasmin Hussain, Aseel Abdalla, Stephanie S Yee, Zev A Binder, Donald M O’Rourke, Steven Brem, Arati S Desai, Ronald Wolf, Erica L Carpenter, Stephen J Bagley

**Affiliations:** 1 Department of Radiology, Perelman School of Medicine at the University of Pennsylvania, Philadelphia, Pennsylvania, USA; 2 Glioblastoma Translational Center of Excellence, Abramson Cancer Center, University of Pennsylvania, Philadelphia, Pennsylvania, USA; 3 Department of Pathology and Laboratory Medicine, Perelman School of Medicine at the University of Pennsylvania, Philadelphia, Pennsylvania, USA; 4 Division of Hematology/Oncology, Perelman School of Medicine at the University of Pennsylvania, Philadelphia, Pennsylvania, USA; 5 Department of Neurosurgery, Perelman School of Medicine at the University of Pennsylvania, Philadelphia, Pennsylvania, USA; 6 Abramson Cancer Center, University of Pennsylvania, Philadelphia, Pennsylvania, USA

**Keywords:** cfDNA, ctDNA, glioblastoma, histology, MRI

## Abstract

**Background:**

Plasma cell-free DNA (cfDNA) concentration is lower in glioblastoma (GBM) compared to other solid tumors, which can lead to low circulating tumor DNA (ctDNA) detection. In this study, we investigated the relationship between multimodality magnetic resonance imaging (MRI) and histopathologic features with plasma cfDNA concentration and ctDNA detection in patients with treatment-naive GBM.

**Methods:**

We analyzed plasma cfDNA concentration, MRI scans, and tumor histopathology from 42 adult patients with newly diagnosed GBM. Linear regression analysis was used to examine the relationship of plasma cfDNA concentration before surgery to imaging and histopathologic characteristics. In a subset of patients, imaging and histopathologic metrics were also compared between patients with and without a detected tumor somatic mutation.

**Results:**

Tumor volume with elevated (>1.5 times contralateral white matter) rate transfer constant (*K*_ep_, a surrogate of blood–brain barrier [BBB] permeability) was independently associated with plasma cfDNA concentration (*P* = .001). Histopathologic characteristics independently associated with plasma cfDNA concentration included CD68+ macrophage density (*P* = .01) and size of tumor vessels (*P* = .01). Patients with higher (grade ≥3) perivascular CD68+ macrophage density had lower volume transfer constant (*K*_trans_, *P* = .01) compared to those with lower perivascular CD68+ macrophage density. Detection of at least 1 somatic mutation in plasma cfDNA was associated with significantly lower perivascular CD68+ macrophages (*P* = .01).

**Conclusions:**

Metrics of BBB disruption and quantity and distribution of tumor-associated macrophages are associated with plasma cfDNA concentration and ctDNA detection in GBM patients. These findings represent an important step in understanding the factors that determine plasma cfDNA concentration and ctDNA detection.

**Key Points**

- In patients with treatment-naive GBM, tumor volume with elevated metrics of BBB disruption is independently associated with higher plasma cfDNA concentration.
- High perivascular CD68+ macrophage density is associated with lower BBB permeability and low detection rate of somatic mutations in plasma specimens.

**Importance of the Study**

Liquid biopsy, particularly the analysis of plasma cell-free DNA (cfDNA) and circulating tumor DNA (ctDNA), has emerged as a minimally invasive tool for molecular profiling and tumor monitoring in clinical oncology. In glioblastoma (GBM), however, cfDNA concentration is lower compared to other solid tumors, which can lead to low ctDNA detection. Improved understanding of the factors that drive cfDNA concentration and ctDNA detection in GBM may lead to more efficient use of liquid biopsy and predict which subset of patients may benefit from liquid biopsy. In this study, we demonstrated that in patients with treatment-naive GBM, metrics of BBB disruption and quantity and distribution of tumor-associated macrophages are associated with plasma cfDNA concentration and ctDNA detection. These findings represent an important step in understanding the factors that determine plasma cfDNA concentration and ctDNA detection, and hence the potential clinical utility of plasma-based liquid biopsy, in patients with GBM.

Liquid biopsy refers to the collection of any bodily fluid from cancer patients for analysis of tumor-derived material that has been shed into the fluid. Several blood-based techniques such as circulating tumor cells, cell-free DNA (cfDNA), and circulating extracellular vesicles have been successfully used in different tumors for prognostication, tumor response assessment, and molecular profiling.^1,2^

Plasma cfDNA refers to fragmented DNA found in the non-cellular component of the blood. In cancer patients, a portion of cfDNA is derived from tumor cells, which is referred to as circulating tumor DNA (ctDNA).^3^ ctDNA is thought to be released from cancer cells into the bloodstream secondary to cancer cell necrosis or apoptosis,^3^ with some ctDNA generated by engulfment of tumor material by macrophages with subsequent digestion of tumor DNA fragments.^4^ The ability to analyze tumor-derived material from a routine blood draw is especially attractive in brain tumors, such as glioblastoma (GBM), given the difficulty in access and the morbidity associated with surgical biopsy. However, due to lower levels of tumor-derived material in the blood of patients with GBM compared to other solid tumors, the use of liquid biopsy in GBM has been relatively limited to date.

In a recent study, we demonstrated that total plasma cfDNA concentration tended to rise prior to or concurrently with radiographic tumor progression in patients undergoing standard chemoradiotherapy for newly diagnosed GBM and was closely correlated with radiographic tumor burden at certain time points, such as the first post-radiation MRI scan.^5^ However, plasma cfDNA concentration was not correlated with radiographic tumor burden preoperatively. This discrepancy indicates that there are a variety of other factors that contribute to cfDNA release into the circulation and that volumetric tumor measurements cannot adequately address the interplay between tumor biology and cfDNA concentration. In addition, tumor-derived plasma cfDNA (ctDNA) concentration is lower in GBM compared to other solid tumors, which may indicate that physical obstacles such as the blood–brain barrier (BBB) limit ctDNA release into the systemic circulation.^6^

In this post-hoc analysis of our original plasma cfDNA study in GBM, we examined relationships between plasma cfDNA concentration in patients with treatment-naive GBM and imaging measures of tumor cellularity, vascularity, and BBB disruption as well as histopathological assessment of tumoral vessel size and macrophage infiltration. We additionally compared histologic features and imaging measures between patients with and without the detection of somatic mutations in plasma specimens. We hypothesized that higher plasma cfDNA concentration would be related to higher tumor cellularity, perfusion, BBB disruption, and vessel size. In addition, given that macrophages and microglia are a major population of the non-neoplastic cells in GBM tumor microenvironment,^7^ we hypothesized that macrophage density and distribution would affect the plasma cfDNA concentration and ctDNA detection.

## Materials and Methods

### Study Design and Patient Population

We analyzed MRI scans and tumor samples from 42 adult patients with newly diagnosed GBM who were previously enrolled in a prospective cohort study at our institution.^5^ Patients were screened from the Neurosurgery services at the Hospital of the University of Pennsylvania on the basis of having an MRI scan suspicious for high-grade glioma and a plan for a diagnostic and/or therapeutic surgical procedure. Subjects who provided informed consent had preoperative whole-blood samples collected in the operating room immediately prior to surgery. In addition, baseline demographic and clinical/laboratory variables were collected, including age, sex, and serum creatinine for calculation of glomerular filtration rate. Following initial blood collection, patients were continued on the study if histopathology confirmed a diagnosis of GBM. This study was approved by the Institutional Review Board of the University of Pennsylvania.

### Specimen Collection and Plasma Isolation

Whole-blood samples were collected in Streck cfDNA blood collection tubes and processed to plasma and frozen within 96 h of the draw. Whole blood was centrifuged at 1600 × *g* for 10 min, the plasma supernatant was isolated and centrifuged twice at 4122 × *g* for 15 min (swinging bucket, break-off), aliquoted, and frozen at −80°C for future use. cfDNA extraction and quantification were performed as previously described.^5^

### Image Acquisition

Brain MRI was performed in all subjects including volumetric axial T1-weighted 3D MPRAGE (TR/TE/TI = 1760/3.1/950 ms, 192 × 256 matrix size, 1-mm section thickness) before and after contrast, as well as axial FLAIR (TR/TE/TI = 9420/141/2500 ms, 3-mm section thickness). Advanced imaging sequences were acquired in a subset of patients, all of whom were imaged on a 3T MRI unit (Magnetom TrioTim, Magnetom Skyra, Magnetom Vida; Siemens) using a 12-channel phased-array head coil. These included diffusion tensor imaging (DTI) (*n* = 30), dynamic contrast-enhanced (DCE) perfusion (*n* = 30), and dynamic susceptibility contrast (DSC) perfusion (*n* = 29).

DTI data were acquired in 30 noncollinear/noncoplanar directions with a single‐shot spin‐echo, echo‐planar read‐out sequence with parallel imaging using generalized autocalibrating partially parallel acquisition with an acceleration factor of 2. The sequence parameters were as follows: TR/TE = 5000/86 ms, NEX = 3, field of view (FOV) = 22 × 22 cm^2^, matrix size = 128 × 128, in‐plane resolution = 1.72 × 1.72 mm^2^; slice thickness = 3 mm; *b* = 0, 1000 s/mm^2^, number of slices = 40.

DSC perfusion imaging was performed with T2*‐weighted gradient‐echo, echo-planar imaging (TR/TE = 2000/45 ms; FOV = 22 × 22 cm^2^; matrix size = 128 × 128; in‐plane resolution = 1.72 × 1.72 mm^2^; slice thickness = 3 mm; BW = 1346 Hz/pixel; flip angle = 90°; EPI factor = 128; echo spacing = 0.83. Forty‐five sequential measurements were acquired for each section. DCE imaging was performed with a dynamic gradient-echo T1-weighted sequence (TR = 4.8 ms; TE = 1.47 ms; slice thickness = 3.5 mm; FOV = 22 × 22 cm^2^; matrix size = 256 × 256). For DCE imaging, a bolus of contrast agent was injected with a dose of 0.1 mmol/kg, which also served as a preload dose for DSC imaging to reduce the effect of contrast agent leakage on relative cerebral blood volume (rCBV) measurements followed by another similar dose for DSC acquisition.

### Image Analysis

**Fig. 1:**
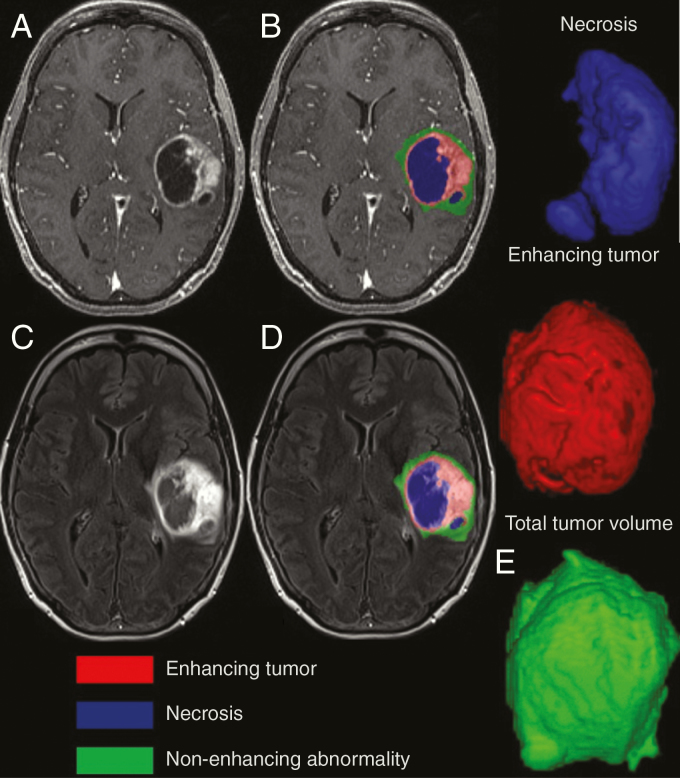
Tumor segmentation in a patient with glioblastoma. Necrosis, enhancing and non-enhancing FLAIR signal abnormality on pre- and post-contrast T1 (A and B) and FLAIR (C and D). (E) The 3D representation of necrosis, enhancing, and total tumor volumes.

Regions of abnormal contrast enhancement, necrosis, and non-enhancing FLAIR signal intensity were segmented using a semi-automated segmentation tool (ITK-SNAP)^8^ followed by manual editing by 2 board-certified neuroradiologists (S.A.N. and J.B.W.) who were blinded to cfDNA values (Figure 1). DTI processing was performed with FSL^9^ and included removal of non-brain tissue as well as correction for motion and eddy currents. Diffusion data were then fit to the tensor model, and whole-brain maps of apparent diffusion coefficient (ADC) maps were used in subsequent analysis. DWI-derived ADC has been shown to inversely correlate with tumor cellularity in gliomas.^10^

DCE MRI was used to assess BBB integrity and calculate quantitative parameters of tumor microcirculation. *K*_trans_ is the volume transfer constant between the plasma and extravascular extracellular space (EES), and *V*_e_ is the EES per unit volume of tissue^11,12^; both *K*_trans_ and *V*_e_ are considered to reflect BBB permeability.^13^*V*_p_ is the blood plasma volume per unit volume of tissue^13^ and *K*_ep_ represents the washout rate constant of contrast agent from the EES to the intravascular space. DCE images were corrected for motion and non-brain tissue was removed using image processing tools available in FSL. DCE data were then analyzed using the extended Toft’s model, as implemented by the ROCKETSHIP software package^14^ in the MATLAB programming environment (2014a, MathWorks), to generate maps of plasma volume fraction (*V*p), extracellular volume fracture (*V*_e_), and vascular transfer constant (*K*_trans_). Tissue transfer constant (*K*_ep_) maps were calculated as *K*_ep_ = *K*_trans_/*V*_e_. Due to the inconsistent availability of T1 mapping among subjects, a fixed pre-bolus T1 value (1000 ms) was used to transform signal intensity curves to contrast concentration curves in DCE analysis. Although T1 mapping provides a more direct measure of tissue relaxivity, clinical T1 mapping sequences are susceptible to artifacts and the additional noise introduced by T1 mapping can ultimately reduce the reliability and reproducibility of final DCE parameter estimation.^15^ Previous studies assessing the utility of DCE in glioma imaging have suggested that T1 mapping in comparison to the fixed T1 method does not improve glioma grading^16^ and may be less accurate in differentiating true tumor progression from pseudoprogression in the posttreatment setting.^17^

DSC data were used to generate leakage‐corrected rCBV maps using the γ‐variate function as implemented in NordicICE software (NordicNeuroLab). FSL’s linear registration tool FLIRT was used to co-register each subject’s FLAIR and T1 postcontrast MPRAGE images in high-resolution structural space. Furthermore, FLIRT was used to calculate transformation matrices between each subject’s T1 post-contrast MPRAGE and the mean images from the 4D source DCE, DTI, and DSC data. These matrices were then used to co-register all parameter maps with the anatomical space. rCBV maps were created by normalizing the CBV maps to the mean CBV value in a region of interest placed by a board-certified neuroradiologist (J.B.W.) in normal-appearing white matter from the contralateral hemisphere at the same slice level. ADC and DCE parameter maps were also normalized in this fashion in order to account for potential inter-scanner variability.

Imaging measures used for further statistical analysis included mean values of ADC, rCBV, and DCE metrics across the non-necrotic component of the tumor, as well as the volume of tumor displaying elevated values of DCE metrics, rCBV, and reduced ADC. Elevated DCE metrics and rCBV were defined as greater than 1.5× higher than the mean of contralateral normal-appearing white matter, and reduced ADC was defined as less than 1.5× lower than the mean of contralateral normal-appearing white matter. To further account for the effects of tumor heterogeneity on imaging metrics, we additionally tested the correlation between cfDNA concentration and the 85th percentile value of DCE metrics and rCBV, and the 15th percentile value of ADC derived from the non-necrotic volume of the tumor. In addition, the analysis was repeated using a threshold of 2.5 to define the volume of the tumor with elevated DCE metrics and rCBV and reduced ADC.

### Histopathologic Evaluation

Tumor specimens from initial surgical resection in each of the subjects were assessed by a board-certified neuropathologist (M.P.N.) who was blinded to cfDNA values. Thirty-nine subjects had enough tissue for additional staining. One representative block of tissue for each specimen was stained with hematoxylin and eosin, as well as with the CD68 antibody. The block for analysis was chosen based on how representative it was of the entirely submitted tissue and often represented greater than 50% of the tissue. Unstained slides were pretreated with heat retrieval, epitope retrieval 1, in citrate buffer pH 6.0 (Leica Microsystems) for 20 min. Immunohistochemical staining was performed on the Bond 111 Autostainer with the DAB chromogen and a hematoxylin counterstain. The following factors were quantified in each specimen: CD68+ macrophage density (graded 1–6; minimal to extensive, averaged across the section), perivascular distribution of CD68+ cells (graded 1–6; minimal to extensive), predominant size of tumor vessels (graded 1–6; small to large), variability in tumor vessel size (graded 1–6; minimal to extensive), and morphology of CD68+ myeloid cells (graded 1–6; 1 microglial to 6 epitheloid). The heterogeneity of the location of CD68+ cells within the tumor was captured by the relative abundance throughout the tumor (density) combined with an indication of the perivascular density. Microglia are monocytic cells resident in the brain and have small nuclei with many fine processes, which become elaborate and thickened in the reactive state as seen by CD68 immunohistochemistry. CD68+ cells with epithelioid morphology may represent microglia that have transformed in response to a pathologic process or macrophages that have been recruited to the CNS from the periphery.^18^ The heterogeneity in myeloid morphology was assessed by the morphology scoring. Likewise, the heterogeneity of the vasculature throughout a single block was captured by the combination of the predominant vessel size and the variability in tumor vessel size.

### Next-Generation Sequencing

In a subset of 20 patients who provided additional consent for third-party sequencing of plasma cfDNA, plasma samples obtained prior to initial surgical resection were sequenced by Guardant Health (Guardant360)^5^ to detect single-nucleotide variants, insertion–deletions (indel), fusions, and copy-number amplifications in 77 genes^5^; 19 out of 20 patients had enough tissue for additional staining. The detection of a somatic variant in plasma was correlated with imaging and histologic findings. A detailed description of all disease-associated somatic variants detected in either tumor tissue and/or ctDNA is provided in our previous manuscript.^5^

### Statistical Analysis

Statistical analysis was conducted in the R statistical environment (version 3.6.1; http://www.r-project.org/) for analysis.^19^ Imaging measures were first examined for normality using the Shapiro–Wilk test. As cfDNA concentrations and imaging measures were found to have non-normal distributions, a non-parametric test (Spearman’s correlation) was used to assess the univariate correlation between cfDNA and each imaging measure, as well as with ordinal histopathologic measures. *P*-values obtained from univariate analysis were false discovery rate corrected to account for multiple comparisons. Subsequently, in order to identify imaging and histopathologic measures independently associated with plasma cfDNA level, a stepwise multivariate linear regression analysis was performed using as candidates all imaging and histopathologic variables associated with plasma cfDNA in univariate analysis with an uncorrected *P*-value of <.1.

Finally, histologic variables, *K*_trans_, and *K*_ep_ were compared between patients with and without at least 1 somatic mutation detected in plasma using the Wilcoxon signed-rank test. In addition, the relationship between *K*_trans_ and *K*_ep_ and histologic variables significantly associated with the presence of a somatic mutation were assessed using Spearman’s correlation and the Wilcoxon signed-rank test. All tests were 2-tailed. A *P*-value <.05 was considered statistically significant.

## Results

Forty-two patients with GBM were included in this study (median age = 65, age range: 20–81 years, 19 females, 23 males).^5^ The mean plasma cfDNA level was 13.43 (SD = 10.35) ng/mL. There was no correlation between baseline plasma cfDNA level and total preoperative radiographic tumor volume (the combination of enhancing tumor, non-enhancing core, and peritumoral T2 hyperintensity, *ρ* = 0.24, *P* = .2). There was also no correlation between plasma cfDNA level and contrast-enhancing tumor volume (*ρ* = 0.17, *P* = .2) and necrotic tumor volume (*ρ* = 0.08, *P* = .5), respectively.

**Fig. 2:**
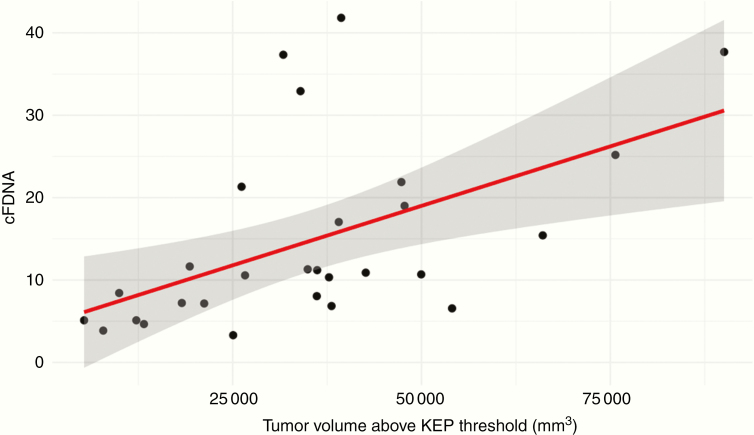
Positive correlation between *K*_ep_ volume (*ρ* = 0.57, *P* = .01) above the threshold and cfDNA concentration.

Mean tumor ADC and DCE metrics (*K*_trans_, *K*_ep_, *V*_e_, *V*_p_), as well as rCBV, were not significantly correlated with cfDNA concentration in univariate analysis (Table 1). Conversely, plasma cfDNA demonstrated a moderate positive correlation with the volume of tumor displaying elevated *K*_trans_ (*ρ* = 0.51, *P* = .03) and elevated *K*_ep_ (*ρ* = 0.57, *P* = .01, Figure 2). Furthermore, there was a weaker positive correlation between plasma cfDNA and volume of tumor displaying elevated rCBV (*ρ* = 0.31, *P* = .12) and elevated *V*_e_ (*ρ* = 0.44, *P* = .08), which were not statistically significant after correcting for multiple comparisons. Plasma cfDNA was not significantly correlated with the volume of tumors displaying low ADC. Repeating the analysis with a higher threshold (2.5) for defining tumor volume with elevated DCE metrics, elevated rCBV, and lower ADC did not alter the statistically significant correlations from the primary analysis. Furthermore, in supplemental analysis 85th percentile measures of DCE metrics and rCBV and the 15th percentile of ADC were not significantly correlated with cfDNA.

**Table 1:** In Univariate Analysis of Imaging and Histopathologic Variables, Volume-*K*_trans_, Volume *K*_ep_, and Size of Tumor Vessels Were Significantly Correlated With cfDNA Concentration

Variables	Univariate Analysis			Multivariate Analysis
	*ρ*	*P*	*P*-adjusted	*P*
Volume-*K*_trans_	0.514	.005	**.034**	—
Volume-V_p_	0.311	.106	.202	—
Volume-V_e_	0.442	.019	.087	—
Volume-rCBV	0.316	.041	.123	—
Volume-ADC	0.210	.255	.328	—
Volume-*K*_ep_	0.571	.001	**.016**	**.001**
ADC-mean	0.028	.882	.885	—
rCBV-mean	0.295	.112	.202	—
*V*_p_-mean	−0.226	.236	.328	—
*V*_e_-mean	−0.109	.570	.642	—
*K*_trans_-mean	0.028	.885	.885	—
*K*_ep_-mean	0.137	.475	.570	—
Total tumor volume	0.240	.124	.204	—
CD68 density	0.346	.030	.110	**.011**
Morphology of CD68 cells	0.262	.106	.202	—
PVD of CD68 cells	0.187	.251	.328	—
Size of tumor vessels	0.591	.000	**.001**	**.012**
Variability in tumor vessel size	0.281	.082	.202	—

On multivariate analysis, volume-*K*_ep_, CD68 density, and size of tumor vessels were independently associated with plasma cfDNA concentration. *K*_ep_ = rate transfer constant; *K*_trans_ = volume transfer constant, *V*_p_ = blood plasma volume per unit volume of tissue, *V*_e_ = extravascular extracellular space per unit volume of tissue, rCBV = relative cerebral blood volume, ADC = apparent diffusion coefficient, cfDNA = cell-free DNA, PVD = perivascular distribution.

Bold indicates significant p values.

**Fig. 3:**
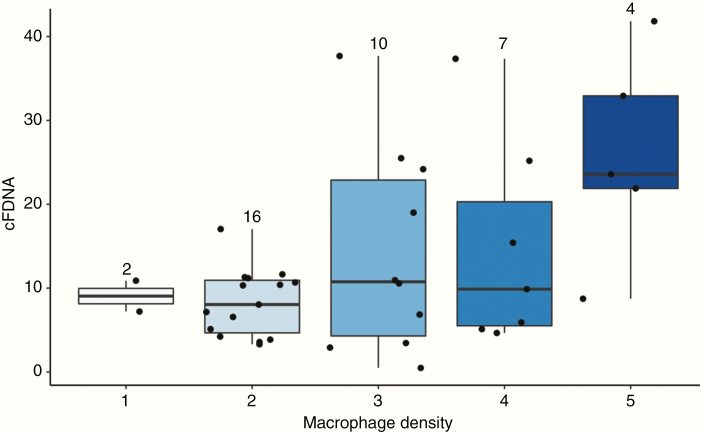
Positive correlation between CD68 density (*ρ* = 0.34) and cfDNA concentration.

**Fig. 4:**
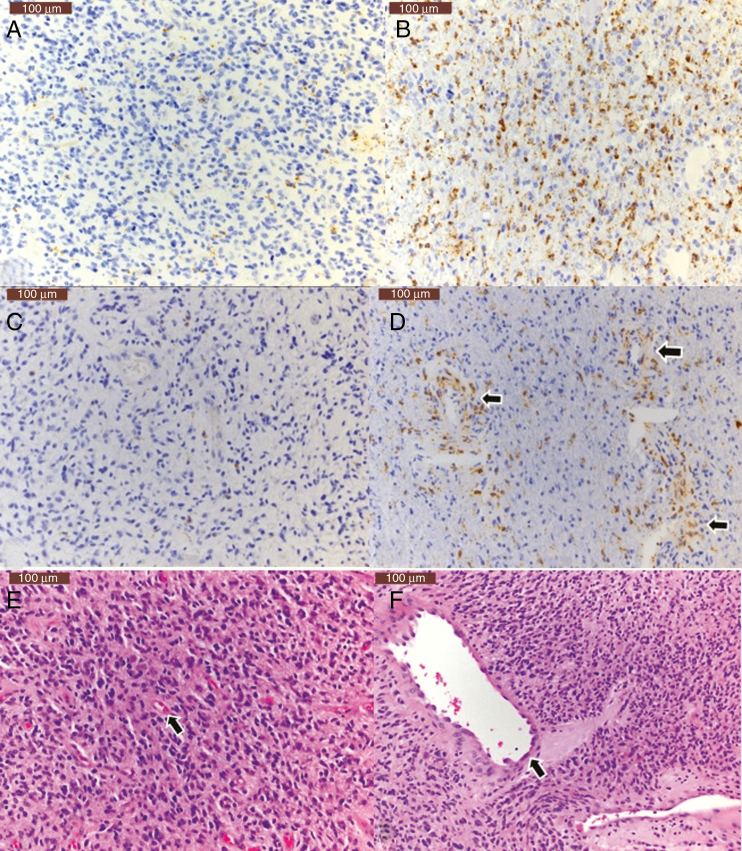
Immunohistochemical assessment of macrophage density with CD68 staining demonstrates tumors with low (A) and high (B) CD68-positive cells. C and D demonstrate low (C) and high (arrows, D) density of perivascular CD68-positive cells. Hematoxylin and eosin stained sections demonstrate tumors with small (E, arrow) and large (F, arrow) size of vessels. Scale bar: 100 µ, upper left.

Regarding histopathologic parameters, the size of tumor vessels was moderately correlated with cfDNA concentration. (*ρ* = 0.59, *P* = .001). There was a weaker positive correlation between CD68+ macrophage density and cfDNA concentration, which was not statistically significant after correcting for multiple comparisons (*ρ* = 0.34, *P* = .11) (Figures 3 and 4). Other histopathological variables, including myeloid cell morphology (*P* = .20), perivascular distribution of macrophages (*P* = .32), and variation in tumor vessel size (*P* = .20), were not significantly correlated with cfDNA concentration (Table 1).

In the stepwise multivariate analysis, the volume of tumor with elevated *K*_ep_ (*P* = .001), CD68+ macrophage density (*P* = .01), and size of tumor vessels (*P* = .01) were found to be independently associated with plasma cfDNA concentration (adjusted *R*^2^ of the final model = 0.62) (Table 1).

Among the 19 patients who had plasma that was collected preoperatively and sequenced by targeted next-generation sequencing, 10 patients (53%) had at least 1 somatic mutation detected. The mean allele fraction of somatic alterations detected in the plasma was 0.95%. Detection of at least 1 somatic mutation in plasma cfDNA was associated with significantly lower perivascular CD68+ macrophages (*P* = .01). Other histopathological variables, including CD68+ macrophage density (*P* = .22) myeloid cell morphology (*P* = .71), tumor vessel size (0.33), and variation in tumor vessel size (*P* = .19), were not significantly associated with detection of at least 1 somatic mutation. Twenty-seven patients had both DCE permeability imaging and histologic data. Among these patients, there were weak negative correlations between mean *K*_trans_ (*ρ* = −0.34, *P* = .08), *K*_ep_ (*ρ* = −0.21, *P* = .28) and perivascular CD68+ macrophage density, which did not reach statistical significance. Finally in order to determine the relationship of perivascular CD68+ macrophage density and BBB permeability, BBB permeability metrics were compared based on perivascular CD68+ macrophage density, which demonstrated that patients with higher (grade ≥3) perivascular CD68+ macrophage density (*n* = 10) had lower *K*_trans_ (*P* = .01, mean normalized *K*_trans_ 3.28 vs 10.76) and tended to have lower *K*_ep_ (*P* = .07, mean normalized *K*_ep_ 2.96 vs 9.69) compared to those with lower perivascular CD68+ macrophage density (*n* = 17).

## Discussion

In this study, we demonstrated that MRI imaging metrics reflecting BBB disruption are associated with plasma cfDNA concentration in patients with newly diagnosed GBM. Specifically, tumor volume with elevated *K*_ep_ and *K*_trans_ was positively correlated with plasma cfDNA. We also showed that increased tumor macrophage density and tumor vessel size, as quantified on histopathology, is associated with increased plasma cfDNA concentration. In addition, we demonstrated that high perivascular CD68+ macrophage density is associated with lower BBB permeability and low detection rate of somatic mutations in GBM plasma specimens.

In a recent study, we demonstrated that plasma cfDNA may be an effective prognostic tool and surrogate of tumor burden in newly diagnosed GBM.^5^ In addition, we demonstrated that the detection of somatic alterations in the plasma of patients with GBM is feasible and correlates with decreased overall survival.^5^ However, the low concentration of cfDNA in GBM compared to other solid tumors remains one of the main limitations for expanding its clinical use.^6^ Given this limitation, a better understanding of the factors that lead to higher cfDNA concentration and a higher detection rate of somatic mutations in GBM may improve the selection of patients who could benefit from liquid biopsy.

To our knowledge, this is the first human study to investigate the relationship between DSC and DCE MRI and diffusion-weighted imaging with plasma cfDNA concentration. We did not find a statistically significant correlation between plasma cfDNA and overall tumor volume, nor with normalized mean tumoral rCBV, *K*_trans_, *K*_ep_, *V*_e_, and *V*_p_. There was, however, a positive correlation with the volume of tumors with elevated *K*_ep_ and *K*_trans_, suggesting that shedding of cfDNA into the blood is dependent on a sufficient level of BBB disruption, but not necessarily influenced by further elevations in BBB permeability beyond this threshold. The relationship between imaging features and plasma cfDNA concentration should be interpreted in the context of highly abnormal vasculature in GBM. In a prior study, we did not find a significant correlation between microvascular proliferation and cfDNA concentration in GBM.^5^ In a patient-derived orthotopic xenograft GBM model, Mair et al.^20^ also did not find a significant relationship between microvessel density (measured by CD31 expression as an endothelial cell marker) and ctDNA concentration. In the current study, however, we analyzed the tumor supplying vessels on histopathology and found a moderate correlation between the tumor vessel size and plasma cfDNA concentrations. These findings may indicate that large tumor vasculature would facilitate the transfer of cfDNA from the tumor microenvironment.

We also found a positive correlation between tumor macrophage density and plasma cfDNA concentration. Previous studies have shown the important role of macrophages in the release of DNA from apoptotic and necrotic cells.^21^ Based on the size of the DNA released, Diehl et al.^22^ proposed that the mutant DNA fragments found in the circulation of patients with colorectal tumors were derived from necrotic neoplastic cells that had been engulfed by macrophages. Hohaus et al.^23^ studied cfDNA in patients with Hodgkin’s lymphoma (HL) and diffuse large B-cell non-Hodgkin’s lymphoma (DLBCL) and demonstrated that patients with HL had similar cfDNA concentration compared to DLBCL despite much smaller fraction of tumor cells, which was attributed to the massive inflammatory infiltrates in HL that consist of a spectrum of different immune cells including tumor-associated macrophages (TAMs).^24^ Given that macrophages and microglia are a major population of the non-neoplastic cells in GBM and comprise as many as 30–50% of the cells in GBM tumor microenvironment,^7^ it is not surprising that there is an association between macrophage density and plasma cfDNA concentration in GBM patients. To our knowledge, this is the first study to investigate the relationship of perivascular TAMs with cfDNA and ctDNA in a solid tumor. The higher detection rate of somatic mutations in patients with lower perivascular TAMs could be justified by the effect of perivascular macrophages in limiting vascular permeability,^25^ which was also confirmed with our imaging data.

This study has several limitations. The sample size is relatively small noting that this is a single-center, post-hoc analysis. Thus, the results of this study should be interpreted with caution and need to be confirmed by a validation cohort and future larger studies. In addition, histopathologic and immunohistochemical analysis was performed on a single block of tissue for each case, and although the block was chosen to best represent the entire specimen, sampling error in these heterogeneous GBM cannot be excluded. We have an ongoing study at our institution and plan to validate these results in an independent cohort. Nonetheless, our initial results provide the first proof of principle regarding the relationships between MRI metrics of BBB permeability and histopathologic characteristics with plasma cfDNA and ctDNA in human GBM.

In conclusion, in patients with treatment-naive GBM, tumor volume with elevated metrics of BBB disruption is independently associated with higher plasma cfDNA concentration. Tumor macrophage density and tumor vessel size are also independently associated with plasma cfDNA concentration. In addition, high perivascular CD68+ macrophage density is associated with lower BBB permeability and low detection rate of somatic mutations in plasma specimens. This study is the first step toward a better understanding of the factors that influence plasma cfDNA concentration and somatic mutation detection in GBM. Future studies are warranted to investigate the clinical utility of these findings to predict which subset of patients may benefit from liquid biopsy.
